# The Role of Motivation and Physical Self-Concept in Accomplishing Physical Activity in Primary School Children

**DOI:** 10.3390/sports11090173

**Published:** 2023-09-05

**Authors:** Slobodan Pavlović, Vladan Pelemiš, Jovan Marković, Marko Dimitrijević, Marko Badrić, Sabolč Halaši, Ivko Nikolić, Nebojša Čokorilo

**Affiliations:** 1 Faculty of Education, University of Kragujevac, 34000 Kragujevac, Serbia; slobodan.b.pavlovic@gmail.com (S.P.); jovo.markovic.72@gmail.com (J.M.); 2 Faculty of Teacher Education, University of Belgrade, 11000 Belgrade, Serbia; ivko.nikolic@uf.bg.ac.rs; 3 Department of Physiology, Faculty of Medical Sciences Kragujevac, University of Kragujevac, 34000 Kragujevac, Serbia; dimitrijevic_marko@yahoo.com; 4 Faculty of Teacher Education, University of Zagreb, 10000 Zagreb, Croatia; marko.badric@gmail.com; 5 Faculty of Teacher Education in the Hungarian Language, University of Novi Sad, 21000 Novi Sad, Serbia; halasi.sabolc@gmail.com; 6 Faculty of Sport, Union University-Nikola Tesla, 11000 Belgrade, Serbia; nebojsa.cokorilo@fzs.edu.rs

**Keywords:** physical activity, physical self-concept, motivation, PE classes

## Abstract

Background: The goal of this research is to identify correlations of motivation and physical self-concept with physical activity among students of younger school age, as well as the level of prediction of motivation and physical self-concept with physical activity of students in physical education classes. Methods: The sample of respondents consisted of 411 students of the third and fourth grades from the territory of the Zlatibor district. A modified Self-Regulation Questionnaire was used to assess students’ motivational orientations, while appropriate subscales of the Self-perception Profile for Children measuring instrument were used to assess physical self-concept. Physical activity is shown as volume and intensive physical activity (vigorous physical activity (VPA)), measured with a Suunto memory belt pedometer and heart-rate monitor. Results: Boys’ motivational predictor variables accounted for 14% (volume) and 28% (VPA) of their physical activity in class, with intrinsic motivation, introjective regulation (just for the level of physical activity), and identified regulation as the most important determinants of physical activity. For girls, the identified regulation variable (from the system of motivational predictor variables) was shown to be the primary predictor variable on both criterion variables (R2 = 0.34 and 0.36). Conclusion: The teaching of physical education for students of younger school age should be conceived by creating a motivational climate, in order to encourage physical activity.

## 1. Introduction

Physical exercise has significant favorable impacts on children’s and adolescents’ health, since it is a complex multifactorial behavior influenced by the external environment and biological elements [1,2]. However, the high prevalence of obesity and amotivation, as well as the declining participation of children and adolescents in physical activity [3], raises serious concerns [4,5,6,7]. There are two levels to the insufficient physical exercise: the first one pertains to physical education lessons at school, while the second one pertains to extracurricular activities. According to the current survey of the World Health Organization (WHO) for the year 2016, about 340 million students of preschool, primary school, and adolescent age were obese or were obese as children, while in 2020 about 39 million children in the world under 5 years of age were categorized as obese [8]. Particularly at younger school age when children are in a sensitive period for the development of motor skills, fundamental motivational processes and physical self-concept—which relate to different levels of children’s physical activity—are of great importance for improving health and physical efficiency [9,10]). Not only can a student’s motivation affect their degree of accomplishment, but the motivation to learn and participate in class itself also acts as an important objective of the teaching process [11]. By fostering an environment that encourages maximal student participation, the quality of teaching can be greatly enhanced by understanding students’ motivations.

Self-determination theory [12] was used as a reference theoretical framework in the interpretation of the role and predictive significance of student motivation in physical education. The theory of self-determination introduces the concept of the so-called continuum of self-determination of behavior, where between amotivation (i.e., loss of internal and external motivation) and internal motivation at the endpoints there are behavioral regulations of increasing levels of self-determination, i.e., different types of external motivation. The theory of self-determination specifies multiple internal and external sources of motivation, i.e., it recognizes the interaction that takes place between intrapersonal, interpersonal, and external factors [13]. The end result of this interaction is the development of autonomous self-regulation that promotes voluntary actions. This interaction is evident in the individual’s attempts to incorporate values, regulatory mechanisms, and experiences from the environment into the self [14]. In contrast to other motivational theories, the self-determination theory introduces the notion of the so-called continuum of self-determination of behavior, where there are behavioral regulations of increasing levels of self-determination between amotivation and internal motivation at the end points.

In an empirical study, three different types of motivation among students in physical education were discovered, relying on the self-determination theory [15]. This cross-sectional study, in a sample of 428 British students aged 14 to 16 years, produced three motivational profiles of students: the “self-determined profile”, the “moderate motivation profile”, and the “controlled motivation/amotivation profile”. The first type includes students with a high level of intrinsic motivation, and these students are characterized by satisfaction, commitment, enjoyment of teaching, and team spirit. These accounted for about 45% of the students. Almost 40% of the students, in the second group, had average scores across all motivation variables. The third group, which made up the smallest proportion of students—about 15%—was distinguished by a low level of self-determined motivation. Physical education motivation varies widely in both intensity and focus. For some students, physical education is their favorite part of the school day, while for others it is the main cause of stress and a reason for missing school [16].

The sources of inspiration can vary, starting with classmates [17], teachers [18], and others. The decline in student participation in physical education lessons over time poses a serious challenge for the instruction being provided. There is a substantial decline in pupils’ attendance at physical education classes during the adolescent years, particularly among female students [19]. Moreover, it was noticed that some students showed little interest in classes, while others skipped physical education altogether [20]. These authors claim that one of the causes of decreased participation in physical education classes is due to students’ bad experiences, including their sentiments of boredom, incompetence, and poor peer judgment.

Self-concept is important in daily life and, hence, in school—particularly in physical education. From the standpoint of many psychological disciplines, having a positive self-concept is important and serves as a good developmental starting point. Instead of being seen as merely a desirable goal, a good self-concept should be highlighted as a crucial component in achieving other desirable outcomes, such as academic success [21]. According to the model of Shavelson et al. [22], self-concept is a hierarchical structure with the general self-concept at the top and the academic and non-academic self-concept at the bottom. Academic and non-academic self-concepts are further distinguished, and the non-academic self-concept is where social, emotional, and physical self-concepts are positioned with matching lower-level components. Self-perception of competence in a certain domain has a substantial motivational component: those who believe that they are competent in that domain are more persistent and show more interest in it [23]. Children who place a high value on their athletic skills are more internally driven to practice, whereas children who place a lower value on their athletic skills are either less likely to participate in sports or are more externally motivated. Self-evaluation in the physical domain is a significant predictor of future sports-related behavior [24] since it is substantially correlated with the decision to engage in physical activity and persistence in doing so [25]. Self-concept—especially self-evaluation of sports competence—and physical activity have a reciprocal relationship: physical activity can help to improve one’s physical self-concept by enhancing one’s movement skills, but physical self-concept can also affect behavior related to physical activity [26].

The aim of the current research was to determine the correlations between motivation, physical self-concept, and physical activity in primary school children, arising from the predictive importance of motivation and physical self-concept in the teaching of physical education.

In accordance with the set goals of the research, hypotheses emerged indicating that there is a significant positive connection between physical self-concept and self-determined motivation to participate in physical education classes as a predictor of the physical activity of students in physical education classes.

## 2. Materials and Methods

### 2.1. Participants

Using a random sampling method, this research included a total of 411 third- (N = 218) and fourth-grade (N = 193) pupils from nine primary schools (average age 11.4 years, SD 0.72) in the Zlatibor district. There were 184 girls (44.8%) (mean body height 145.2 cm, body weight 42.38 kg, and BMI 20.10) and 227 boys (55.2%) (mean body height 144.6 cm, body weight 39.95 kg, and BMI 19.10). If we start from the fact that the population of third- and fourth-grade students in the district is about 12,000, it can be said that this sample of respondents is representative. The participants frequently participated in the curriculum-provided physical education classes (three times per week). All measurements and assessments were carried out in accordance with the principles of the Helsinki Declaration and after obtaining the consent of the Ethics Committee of the Faculty of Education of the University of Kragujevac under number 26/122021.

### 2.2. Measurement

#### 2.2.1. Motivation

A modified Self-Regulation Questionnaire [27] was used to assess students’ motivational orientations, with adaptations by Goudas et al. [28]. This questionnaire, which has been frequently used among students, can be regarded as appropriate for the research purposes [29,30,31]. We have obtained an average high degree of apprehension (Cronbach’s alpha; α 0.86). Students in the classrooms completed the questionnaire after receiving information on how to do so in advance. The survey has five subscales, each with three or four items, and the items of each subscale are alternated:

There are 3 items on the amotivation subscale: (1) I don’t really understand why I take physical education, (2) I don’t see the need for physical education classes, and (3) I feel like physical education lessons are a waste of time.

- There are 4 items in the external regulation subscale: (1) physical education is required of me, (2) it is something I must do since it is expected of me, (3) it keeps the teacher from yelling at me, and (4) it is something everyone must do for health reasons.
- The introjected regulation subscale comprises the following 4 items: (1) I want my physical education teacher to think I am a good student, (2) I would feel horrible about myself if I didn’t participate in physical education, (3) I would feel ashamed, and (4) I would feel guilty if I didn’t participate in physical education.
- There are 4 elements in the identified regulation subscale: (1) I want to learn new sports skills, (2) I want to succeed in physical education, (3) I want to advance in my physical education classes, and (4) because I think physical education is good for my health.
- The intrinsic motivation subscale has 3 items: (1) because I enjoy learning new sports skills, (2) because it is interesting and exciting for me to practice in physical education class, and (3) because practicing in physical education class is fun.

#### 2.2.2. Physical Self-Concept

The Self-perception Profile for Children was used to measure physical self-concept using the appropriate subscales (SPPC) [32]. The SPPC is a measurement instrument with established metric qualities that has been utilized extensively in related research for a long time [33,34,35,36], so it can be regarded as a suitable measurement tool for this research. In a similar study [37], an average high degree of apprehension was proven (Cronbach’s alpha; α 0.82).

Moreover, the purpose of this measurement tool is to evaluate five distinct, focused areas of children’s self-evaluation, in addition to overall self-evaluation (six subscales in total). Physical appearance and sports competence are two subscales designed to measure one’s physical self-concept. Each subscale has six items (statements). Each statement is written as a two-part sentence, with the first half referring to the child’s competent behavior and the second part referring to their incompetent behavior (e.g., “Some children are very good at all kinds of sports, but Other children think they are not very good at sports”). The respondent should first choose whether the first or second portion of the statement best represents them, and then they should determine whether that description fully or partially applies to them for the part of the sentence that they have chosen. With 1 denoting the least competence in the observable domain and 4 denoting the greatest, the score on each subscale is calculated as the arithmetic mean of the respondent’s responses to specific statements.

#### 2.2.3. Physical Activity of Students

The physical activity of the students in the physical education class was measured using measurement tools that captured the volume and intensity of the students’ physical activity. A new generation pedometer called CoachGear was used to gauge the levels of physical activity among the children in the physical education class. This device counts the total number of steps taken and calculates the distance traveled, the average speed of movement, and the highest speed possible. The students wore the pedometer attached to their belts, and at the end of the lesson the result was read on the display. The total number of steps performed during the physical education class was used as a criterion for this study.

Using a Suunto memory belt heart-rate monitor, the students’ levels of physical activity during the physical education class were gauged. Age, body mass, and body height were entered into the device prior to application. The gadget was then fastened to the chest region, with an electrode in the center. The device was confirmed to be in use following the sound signal. The data from the instrument were read using a reader and immediately uploaded to the computer at the conclusion of the measurement. The value of the heart rate at rest was recorded by positioning the device on the center of the chest and turning it on. These numbers were used to determine workout intensity zones. Each intensity zone describes the level of load that students experience while participating in physical education class activities. When the value of the heart rate at rest is added to a value that is less than 25% of the heart rate at rest, the upper limit of light physical activity (LPA) is reached. The zone of moderate physical activity (MPA) is then created by adding the resting pulse values and the pulse values between 25 and 50% of the resting pulse, while the zone of vigorous physical activity (VPA) is calculated by adding the resting pulse value and values larger than 50% of the resting pulse. Both measurement instruments showed a high degree of reliability in a similar study [37] (Cronbach’s alpha; α 0.89)

1. Light physical activity (LPA) = heart rate at rest + x < 25% heart rate at rest;
2. Moderate physical activity (MPA) = heart rate at rest + 25% < x < 50% heart rate at rest;
3. Vigorous physical activity (VPA) = heart rate at rest + x > 50% heart rate at rest.

From a theoretical standpoint, a major factor in students’ participation in physical education classes is the amount of time that they spend engaging in vigorous physical activity (VPA). As a result, in subsequent studies, vigorous physical activity (VPA) was chosen as a representative criterion variable, and other intensity zones were ignored.

In the previous period, all of the abovementioned measurement instruments were used in research on the population of students in elementary schools in Serbia, which is where the reliability of these tools comes from.

### 2.3. Procedures

This study was carried out in the 2021–2022 academic year, specifically in the months of September, October, and December. For the three months stated, which comprised the research period, the students’ physical activity was measured while they were in class. The class was divided into four parts (introductory, preparatory, main, and final parts of the class). The students used easy, natural movement patterns to enhance their physical efforts and get ready for the rest of the lesson during the introduction phase. Four different catch-style games were put into practice. The goal of the games was to determine the individual who would have the task of catching one of the students in the shortest possible time, followed by determining an individual who would make a “chain” of caught students, and so on until the last student was caught. One of the activities was performing the mentioned tasks with limited movements (jumping with both legs—barefoot, jumping on one leg (more dominant and less dominant)). The selection of students to start performing the movement task was random, and after that it depended on the rules of the game. In order to prepare for the activities that would come later in the main part of the session, the students engaged in the proper shaping exercises (loosening, stretching, and strengthening exercises) during the lesson’s preparation phase. Activities were carried out in the main portion of the lesson in accordance with the teaching unit that was made available for the specific lesson, and everything was carried out in accordance with the annual curriculum. In order to calm their bodies, lower their heart and respiration rates, and sufficiently prepare them for the end of the lesson and the rest of the school day, the children ran lightly during the final portion of the class. Care was taken to limit the teacher’s direct impact on the students’ level of involvement or physical activity during all of the activities in the physical education class. As a result, suitable techniques and formats for classwork were selected. The focus was on physical activities that let students choose for themselves how active they will be in class (i.e., the teacher does not directly influence their activity).

### 2.4. Statistical Analysis

The statistical data analysis tool SPSS 20 was used to conduct statistical analyses (v21.0., SPSS Inc., Chicago, IL, USA). Basic descriptive statistics were determined for all variables: arithmetic mean (M), standard deviation (SD), and coefficient of variation (CV). Associations between variables were calculated using Pearson’s correlation coefficient, which was a prerequisite for the further use of regression analysis. Multiple regression analysis was used to determine the relationship between the set of predictor variables and the criterion variable. The physical activity of students in physical education class, measured by volume (number of steps) and intensity, represented the criterion variable in the study. The system of predictor variables included students’ physical self-concept (i.e., factors of physical appearance and sports ability) and motivation to participate in physical education classes. After stating that the entire system of predictor variables had a significant connection with the criterion, based on standardized regression coefficients (β) and their significance, predictive values were determined. A significance level of *p* ≤ 0.05 was used for significance testing.

## 3. Results

Table 1 displays descriptive statistics for predictor variables (i.e., physical self-concept, motivation to participate in physical education class), as well as descriptive statistics of criterion variables (i.e., volume of physical activity in physical education class and level of intensive physical activity in physical education class), for boys and girls. Boys and girls on average performed better in physical appearance (boys 3.67; girls 3.42) in comparison to sports competence (boys 2.53; girls 2.82). When it comes to the motivation to participate in the physical education class, based on the values of the means, it is apparent that the boys showed the best result on the subscale intrinsic motivation (4.25). This was followed by the results for the subscales of introjected regulation (4.01), identified regulation (3.52), external regulation (3.21), and amotivation (3.03). Similar to boys, females performed best on average in the evaluation of intrinsic motivation (4.12), followed by introjected regulation (3.89) and identified regulation (3.8). Girls reported greater scores of amotivation (3.64) and external regulation (3.49) compared to boys.

Boys averaged 2451 steps and 23.5 min of vigorous physical activity, according to descriptive statistics of the criterion variables, whereas girls were somewhat less active (2285 steps; 21.7 min spent in intense physical activity).

Table 2 shows the values and levels of statistical significance of correlations between the predictor and criterion variables in boys. The criterion variable vigorous physical activity (VPA) achieved the highest number of statistically significant correlations with predictor variables (intrinsic motivation 0.88 **, introjective regulation 0.81 **, sports competence 0.67 *, and identified regulation 0.62 *). When it comes to the second criterion variable—the volume of physical activity—the highest level of correlation was achieved with the predictor variables intrinsic motivation (0.64 **) and sports competence (0.55 *).

Table 3 demonstrates that while the volume of physical activity is significantly (albeit at a lower level of significance) related only to intrinsic motivation (0.59 *), the intensity of physical activity among girls in physical education class is significantly related to both identified regulation (0.73 **) and intrinsic motivation (0.75 **). Girls showed a higher correlation (0.54 *) between vigorous physical activity and physical appearance compared to boys.

The results of the regression analysis and the predictive capability of the two systems of predictor variables are presented in Table 4. Boys’ motivational predictor variables account for 14% (volume) and 28% (vigorous physical activity) of their physical activity in class. Intrinsic motivation, introjective regulation (just for the level of physical activity), and identified regulation were the most important determinants of physical activity in boys.

Regarding the physical self-concept, sports proficiency emerged as a key predictor of boys’ in-class physical activity in both criterion variables. The results for girls were slightly different; for them, the identified regulation variable (from the system of motivational predictor variables) was shown to be the primary predictor variable on both criterion variables. While physical appearance (from the physical self-concept system of variables) dominated as a predictor on both criterion variables of physical activity in girls, the prediction of intrinsic motivation was only obtained in boys for one criterion variable—the level of physical activity.

## 4. Discussion

This study sought to ascertain the degree and significance of correlations between motivation and physical self-concept—that is, their structural components—and the physical activity of students in physical education class, using a representative sample of 411 third- and fourth-grade students of younger school age. The degrees of motivation system and physical self-concept prediction with respect to students’ physical activity in the physical education class were then established. Two variables were used to indicate physical activity: (1) the volume of physical activity, which was determined by the number of steps taken; and (2) the intensity of physical activity, which was determined by the amount of time spent engaging in vigorous physical activity (VPA).

Summarizing the basic premise of the theory of self-determination on the relationship between internal and external motivation, it is possible to single out six modalities of student motivation for physical activity in physical education classes: (1) amotivation, (2) external regulation, (3) introjected regulation, (4) identified regulation, (5) integrated regulation, and (6) intrinsic motivation. The first modality, the so-called amotivation, is characterized by the lowest degree of autonomy; that is, the student has neither internal nor external motivation to engage in physical activities. The second modality—external regulation—indicates that the physical activity of students is determined exclusively by external factors, such as rewards and punishments.

Introjected regulation is the third modality, and its main feature is that the student finds support for physical activity in theirself, but physical activity itself has an instrumental value, and the student begins to recognize the importance of engaging in physical activities.

The fourth modality—identified regulation—implies that the student begins to consciously value the importance of physical activity as a personally important activity for the purpose of progress, and for the improvement of general and mental health.

Integrated regulation is the most autonomous form of the student’s extrinsic motivation for physical activities, and the student’s behavior is integrated with their needs, value system, and value orientations (e.g., the student regularly engages in physical activities because they believe that cultivating a healthy lifestyle and habits represents one of the highest values). The highest degree of autonomous behavior as an expression of the student’s internal needs is related to the sixth modality—intrinsic motivation—and implies that the student engages in physical activities due to the desire for new knowledge and challenges, because they provide them with satisfaction and joy in themselves (e.g., the student regularly engages in physical activities because they want to expand their knowledge, satisfy their curiosity, and test their own capabilities and endurance).

In general, it was found that boys showed better correlation and predictive significance on the criterion variables than girls did for both the predictor systems and the individual structures within them. Only certain structures of motivation (i.e., identified regulation, introjective regulation, intrinsic motivation) significantly predicted the volume and intensity of physical activity of students of both sexes. In the second tested model, the volume and intensity of physical activity of the students significantly predicted the dimensions related to the self-assessment of sports competence in boys, as well as the assessment of physical appearance in girls. The criterion variable vigorous physical activity had higher correlations and predictions at the same time (VPA). In comparison to the number of steps walked or the distance traveled, achieving vigorous physical activity is a more difficult challenge in practicing physical education. This demonstrates that only students who are more driven may reach and accomplish the more difficult goals of physical education classes [38].

In physical education classes, students’ motivation and physical self-concept are crucial indicators of their physical activity [39]. To ensure that physical education lessons are planned and implemented with purpose and that teachers are aware of what to expect from students, it is crucial to analyze the relationships and influences of the aforementioned systems of predictor variables on the physical activity of students in physical education classes. Students’ physical activity during physical education class is highly connected with higher (or more autonomous) levels of motivation. The physical activity of students in physical education classes was not statistically correlated with amotivation or external regulation, but the criterion variables were statistically correlated with introjective and identified regulation as well as intrinsic motivation. Because children are still in the pre-adolescent stage and do not have a strong need to defend the ego—that is, to avoid punishment and feelings of guilt—this is to be expected at the younger school age. Since the drive to move is an internal and ingrained quality of children, they move without external “burdens” and are active without considering the outcomes of the action (i.e., whether they are deemed successful in what they do by others or peers). To fulfill the fundamental psychological requirements for autonomy, competence, and connection with others, physical education programs should be structured in a way that encourages students’ internal motivation. Self-determined student conduct in physical education classes is linked to a variety of favorable outcomes (psychological, behavioral, and cognitive) and can increase student involvement [40]. Students should be permitted to make decisions and choices during class activities (need for autonomy); however, in order to foster the identified motivation, it is important to explain to them not only what and how they should do, but also why. Physical education classes should be engaging and enjoyable, since they encourage students’ intrinsic motivation [26].

The self-assessment of sports competence showed a significant correlation with both criterion factors of boys’ physical activity in physical education class, as it relates to the second system of predictor variables—physical self-concept. Boys who believed that they were better at sports took more steps and remained longer in the zone of intense load in class, and they were more likely to engage in vigorous physical activity (VPA) after class. It must be remembered that these respondents were younger students with inadequately developed self-evaluation criteria. Additionally, because self-evaluation of sports competence is positively correlated with enjoyment of physical education, it may have a long-term (accumulative or delayed) impacts on the amount of physical activity among children and adolescents [41,42]. In contrast to girls, boys did not show significant correlations between the criterion factors and the predictor system’s physical appearance variable. However, girls reported significant correlations between the physical appearance variable and both criterion variables, and this variable dominated the predictor system for the physical self-concept variable. When participating in physical education in class, girls who felt confident about their looks and thought that this was desirable performed better. These results are to be expected if we consider that strength is not the primary factor in the majority of girl-specific activities (rhythmic gymnastics, volleyball, dance, folk dancing, etc.) [43]. Due to research suggesting that girls’ favorable perceptions of their physical appearance may deteriorate between puberty and adolescence, it is especially important to pay close attention to these findings in girls [44]. As a result, the findings of this study can be utilized to support efforts to stop girls from experiencing issues with physical appearance assessment.

## 5. Limitations

This study was cross-sectional. Therefore, a longitudinal study would be of great importance for further discussion and proposed solutions. Moreover, this study is limited to the specified age group, so it is recommended to conduct this and similar research at other ages, especially in the adolescent period.

## 6. Conclusions

The primary objective of this research was investigating the roles that motivation and physical self-concept play in the physical activity of children of younger school age in physical education classes. We found that the physical activity of both boys and girls depends on motivational predictor variables. Additionally, we identified regulation as the most important determinant of physical activity, and it was different for boys and girls. Therefore, teaching of physical education for students of younger school age should be conceived by creating a motivational climate, in order to encourage physical activity.

In order to achieve a shared objective—the healthy education of children and preparation for an active lifestyle—we must be able to collaborate with parents and the broader community as well as creatively and successfully encouraging students’ participation in physical education classes. Also, there is a need for the development and application of a scale that measures the integrated regulation of students for physical activities, in order to obtain a more complete picture of student motivation in physical education classes.

## Figures and Tables

**Table 1:** Descriptive statistics of criterion and predictor variables in boys and girls.

Variables	BOYS			GIRLS		
	Mean	SD	CV%	Mean	SD	CV%
Physical activity volume (n)	2451	436.29	18.79	2285	345.43	15.53
VPA (min)	25.5	4.67	21.17	21.7	3.52	17.05
Amotivation	3.03	0.69	14.36	3.64	1.67	28.61
External regulation	3.21	2.95	22.15	3.49	3.11	21.49
Introjected regulation	4. 01	4.59	10.54	3.89	4.28	10.11
Identified regulation	3. 52	1.59	9.97	3.61	1.71	7.79
Intrinsic motivation	4.25	0.45	6.52	4.12	0.69	5.38
Sports competence	2.53	0.59	20.48	2.82	0.58	20.64
Physical appearance	3.67	0.59	16.96	3.42	0.58	17.04

Legend: VPA—vigorous physical activity; SD—standard deviation; CV%—coefficient of variation.

**Table 2:** Pearson’s correlation of predictor and criterion variables for boys.

Variables	1	2	3	4	5	6	7	8	9
Amotivation	–								
2. External regulation	0.15	–							
3. Introjected regulation	0.09	0.39 *	–						
4. Identified regulation	0.14	0.48 *	0.73 **	–					
5. Intrinsic motivation	0.11	0.05	0.41 *	0.17	–				
6. Sports competence	0.08	0.26	0.36	0.40	0.59 *	–			
7. Physical appearance	−0.03	0.13	0.19	0.42	0.50 *	0.68 **	–		
8. Physical activity volume	−0.07	0.22	0.08	0.16	0.64 **	0.55 *	0.46	–	
9. VPA	0.33	0.37	0.81 **	0.62 *	0.88 **	0.67 *	0.49	0.85 **	–

Legend: **—correlation at the level of significance 0.01; *—correlation at the level of significance 0.05; VPA—vigorous physical activity.

**Table 3:** Pearson’s correlation of predictor and criterion variables for girls.

Variables	1	2	3	4	5	6	7	8	9
Amotivation	–								
2. External regulation	0.19	–							
3. Introjected regulation	0.08	0.33 *	–						
4. Identified regulation	0.17	0.42 *	0.73 **	–					
5. Intrinsic motivation	0.07	0.02	0.43 *	0.16	–				
6. Sports competence	0.10	0.22	0.29	0.28	0.43	–			
7. Physical appearance	−0.04	0.10	0.08	0.40	0.49 *	0.58 *	–		
8. Physical activity volume	−0.09	0.21	0.05	0.11	0.59 *	0.42	0.46	–	
9. VPA	0.44	0.37	0.50 *	0.71 **	0.75 **	0.39	0.54 *	0.77 **	–

Legend: **—correlation at the level of significance 0.01; *—correlation at the level of significance 0.05; VPA—vigorous physical activity.

**Table 4:** Regression analysis of predictors of students’ physical activity in physical education lessons.

Variables	Physical Activity Volume				VPA			
Sex	Boys		Girls		Boys		Girls	
	r_part_	Beta	r_part_	Beta	r_part_	Beta	r_part_	Beta
Amotivation	−0.08	0.04	−0.11	0.06	0.33	0.07	0.44	0.06
External regulation	0.27	0.12	0.29	0.12	0.37	0.18	0.37	0.11
Introjected regulation	0.12	0.18	0.15	0.10	0.83 **	0.13	0.50 *	0.09
Identified regulation	0.19	0.16	0.22	0.08	0.65 *	0.13	0.71 *	0.08
Intrinsic motivation	0.74 **	0.29	0.71 *	0.14	0.91 **	0.28	0.75 **	0.13
**R**	0.18		0.16		0.12		0.10	
**R^2^**	0.14		0.11		0.28		0.19	
**P**	**0.02**		**0.05**		**0.00**		**0.02**	
Sports competence	0.64 *	0.25	0.43	0.02	0.69 *	0.26	0.39	0.07
Physical appearance	0.48	0.12	0.47	0.06	0.52	0.18	0.54 *	0.11
**R**	0.31		0.29		0.27		0.17	
**R^2^**	0.09		0.08		0.18		0.12	
**P**	**0.04**		**0.13**		**0.00**		**0.05**	

Legend:—r_part_—partial correlation coefficient; Beta—regression beta coefficient; R—multiple correlation coefficient; R^2^—coefficient of determination; P—significance of the multiple correlation coefficient; **—correlation at the level of significance 0.01; *—correlation at the level of significance 0.05.

## Data Availability

The data presented in this study are available on request from the corresponding author.
